# Report of a Peculiar Case*Reported to the Louisville Surgical Society

**Published:** 1897-05

**Authors:** Joseph M. Mathews

**Affiliations:** Professor of Surgery and Clinical Lecturer on Diseases of the Rectum in the Kentucky School of Medicine; Rectologist to the Kentucky School of Medicine Hospital and The Louisville City Hospital, etc., Louisville, Kentucky


### Southern Medical Record.

A MONTHLY JOURNAL OF MEDICINE AND SURGERY.

VOL. XXVII. ATLANTA, GA., MAY, 1897. No. ~~4~~ 5

### Original Articles.

### REPORT OF A PECULIAR CASE.*

BY JOSEPH M. MATHEWS, M. D.

Professor of Surgery and Clinical Lecturer on Diseases of the Rectum in the Kentucky School of Medicine; Rectologist to the Kentucky School of Medicine Hospital and The Louisville City Hospital, etc., Louisville, Kentucky.

Several months ago there came to me from a Southern State, a very distinguished educator, accompanied by his wife. He had an ordinary case of internal hemorrhoids. I operated upon him under chloroform, observing the strictest antiseptic precautions, etc., as I usually do. The method used was the ligature. I saw him every day for several days and dressed the parts myself. After the expiration of two weeks these cases usually leave the Infirmary, but this patient, being rather a delicate man, going east for a vacation, concluded to remain a little longer than that, although I could have pronounced him ready to leave at the end of two weeks. There was no discharge from the rectum, except perhaps a little from the granulating material. He was up walking around.

I then left the case in the hands of my assistant, Dr. Laws, who washed the parts every day, or every other day for a few days, after which his wife simply sponged the anus daily, which was all that was necessary. At the expiration of another week, which would make the third week, they took the train for an Eastern resort. On the train the wife complained of intense pain in her eye. In a few hours the eye was swollen enormously and, they said, as large as a small orange. When

*Reported to the Louisville Surgical Society.

they reached their destination, which was less than eight hours after the pain was noticed, the gentleman sent for a physician, and the physician told him that it was of such importance and character, that he should send, or telegraph, immediately to Pittsburg for an oculist. There was great pain and great swelling of the parts. The oculist answered the summons from Pittsburg, and told him it was a very serious condition, and in questioning them about the history, was told that the gentleman had been operated upon for hemorrhoids about three weeks before. The oculist said then that this woman, his wife, must have gotten some of the discharge, pus from the rectum, in her eye, and that the eye should be immediately taken out, and advised her, I suppose because he did not have any of the necessary instruments with him, to go to Philadelphia. They hurried to Philadelphia, and in the Jefferson College Hospital had a consultation with another oculist, and the gentleman writes me that this oculist also agreed that it must have been pus from the rectum which had destroyed the eye, and they immediately chloroformed the woman and removed the eyeball. They said the other eye was in great danger, therefore the affected eye must come out, and the gentleman stated in his letter, that they thought under careful treatment they could save the other one.

I wrote to the gentleman that I did not believe the statement; in the first place there was no discharge from this man's rectum. After a lapse of three weeks there is none. In the second place, granting that there had been any amount of pus from the rectum, it could not have affected the eye. I can not conceive that there had been pus—more than that—had some of this pus gotten into the woman's eye, that it would have destroyed the ball. There is not in any surgical work on diseases of the rectum extant, an intimation that any such result would occur. There is not in any surgery that is published in this country, or in Great Britain, a single intimation that any such result could occur. There is not in any medical book that was ever written, any direction that the hands of the assistants should be washed, presuming, of course, that they would wash them, as far as that is concerned, after dressing a surgical wound, but I mean of course looking to the point that it would destroy the eyesight.

I believe the solution of the affair is that the woman on the car *en route* to the Eastern resort, in the use of a towel, perhaps, infected her eye with gonorrheal pus. Now, I do not know what investigation was made, microscopically or otherwise, but I can not understand why on the train coming up in an hour or two, this violent inflammation should manifest itself, when she did not handle the part as far as dressing it but two or three times, and that was simply to use a little water for the sake of cleanliness.

Of course the patient regrets that his wife did not wash her hands, and what I want to know is this: Is it possible for such a thing to occur? I know a great deal has been said about the colon bacillus, etc., but never in all my experience, have I had anything like this occur, but, to the contrary, have had my hands covered with pus, and have undoubtedly wiped my eyes with pus on my hands. I do not believe this is the correct solution. This is my individual opinion, it may or may not be true, and I would like to know what could have produced the loss of this woman's eye.

Dr. W. C. DUGAN: "It is certainly a very unique case. I agree with Dr. Mathews that the rectal trouble had nothing to do with it. In the first place the rectum was not discharging pus; the patient was doing well and in the second week; Dr. Laws had charge of the case for another week, and he left here in good condition. I am satisfied that the rectum at that time was not discharging a character of pus that would produce the condition described about the eye, and there certainly must have been some other cause of the inflammation. Whether we shall ever be able to ascertain just what it was or not, I am certain the rectum had nothing to do with it."

Dr. W. L. RODMAN: "I agree with Dr. Mathews and Dr. Dugan. I do not think infection could have taken place from simple, or what older authorities called laudable pus, coming from the rectum. If it were possible, as Dr. Mathews says, some of us would have seen instances of that kind in nurses or friends, who have administered to patients with suppurative processes. I think the explanation Dr. Mathews has given as to the possibility of infection, is far more rational than the one given by the oculists."

Dr. J. M. RAY: "All of us who practice ophthalmology encounter every once in a while, cases of violent inflammation of the conjunctiva, which it seems this case was, that goes on to destruction of the eye, where we are unable to locate the source of infection. We have all encountered such cases in the female, and I am always careful to have a vaginal examination made. Writers on purulent ophthamia, and various books on eye diseases, name a number of sources of infection, I do not believe experiments have proven that so-called laudable pus, placed in contact with the conjunctival membrane, does produce typical purulent ophthalmia. The fact remains that we do encounter cases of purulent ophthalmia in which we are unable to find the so-called gonococcus of Neisser. I have had a number of such cases, and in the last few months have seen a case of intense purulent conjunctival inflammation in a young girl eight years of age, in which on vaginal examination, it was found that there was a very profuse vaginal discharge. Of course, we could not get any history from the child as to whether there had been gonorrheal infection or not; the mother was inclined to think such a thing impossible, and it was probably a case of vaginitis, non-specific in character. While the eye was saved, at one time there was some doubt whether it could be done, the corneal inflammation became quite severe, led to superficial corneal ulceration, leaving a small scar, but it did not penetrate.

"Dr. Cheatham calls to my mind a case we saw, in which it was impossible to locate the source of infection; the child had quite a serious inflammation of the eye. The father of the child seemed to think that infection came from a dog that the little girl had been playing with. I recall a number of cases of purulent ophthalmia in young girls in which there was present a profuse vaginal discharge. I believe certain cases of leucorrhea are capable of producing simple forms of infection. For that reason it would have been wise to try and locate the cause of the infection, to have examined for a vaginal discharge in the patient of Dr. Mathews. In using a syringe she might have gotten pus on her hands and infected her eye in this way. We know infection occurs in this manner frequently in the male. In these cases we get an intense swelling of the connective tissue of the lid; I have seen the whole side of the face infiltrated down to the angle of the mouth, an intense erysipelatous like swelling. Some of these cases assume a very malignant type and whatever the cause destruction of the eye results in a short time.

"As to danger to the other eye: Danger to the good eye was simply from getting the discharge from the affected eye into it. As to sympathetic ophthalmia, we know that it never develops under three weeks. Three weeks to three months is the usual time after injury or perforation of the eyeball before the other eye begins to show evidences of involvement.

"It seems hardly possible that this patient's eye could have been infected on the train. From Dr. Mathews' statement she was on the train less than fourteen hours, and it is hardly possible for gonorrheal pus to get in the conjunctiva and to develop and destroy the eye in this short time. It takes from twenty-four to forty-eight hours for gonorrheal infection to reach anything like the condition Dr. Mathews describes as being present when the patient reached her destination. Evidently the source of infection was introduced before the patient left Louisville."

DR. WM. CHEATHAM: "I do not believe an oculist of any standing would advise enucleation of the eye under such conditions. I do not see how he could consistently recommend such a course, if it was simply a case of gonorrheal ophthalmia or suppurative conjunctivitis. It might have been panophthalmitis; that would give a prominent eyeball and account for some of the symptoms. In such cases we usually have very little discharge. Without any further history I do not see how we can ascertain the cause of the trouble; whatever it was I do not think the rectum had anything to do with it. We see in tear duct cases where the bone is involved and where there is periostitis pus about the eye, especially abscesses of the tear sac where even the bone is involved have pus flowing back into the eye for weeks and months, yet there may be no true suppuration of the conjunctiva with symptoms such as Dr. Mathews has described. Should she, however, have an abrasion of the cornea, then get pus in the eye, such condition as Dr. Mathews describes might follow from infection through this abrasion, and enucleation be indicated."

Dt. LOUIS FRANK: "I agree in the main with what the previous speakers have said, yet I would suggest that infection in this case may have taken place from the rectum. We know that we may have a rectal gonorrhea, hence infection possibly of gonorrheal origin might have taken place in the rectum. The case as it stands from the report would lead us to believe that infection was from this source. If my memory serves me correctly, the ordinary pus organisms can not infect the eye nor any other tissue unless there is an abrasion where they can find entrance. The organism of gonorrhea is the only one which is capable of producing infection without an abrasion being present, the infection being in the cells themselves, and the infection as we know, is especially prone to occur in the epithelial cells covering the conjunctiva. Upon what grounds the eastern ophthalmologists based their opinion of course I can not say, but without any microscopical or bacteriological examination of the pus, it seems to me they could not positively say that the infection in this case was from an ordinary purulent discharge from the rectum; it would be impossible for it to occur without an abrasion. The woman may have been infected elsewhere, or the husband may have had an old gonorrhea and infection occurred in some way from this."

Dr. I. N. BLOOM: "I desire to call attention to the incompleteness of the history of the case: In the first place the exact nature of the eye trouble with which the woman suffered is in doubt; we know nothing more than the general symptoms as given by Dr. Mathews. In order to complete the case we need an accurate diagnosis and a description as to the extent of the suppurative conjunctivitis, if she suffered with this affection. Second, the condition of the woman is not known. Of course there was no occasion for Dr. Mathews to examine her. Latent or even acute gonorrhea can not be excluded. Third, the surroundings of the woman before she left on this trip. She may have gotten the germs of the disease, probably gonorrhea, from attendants before she left on the train, or she may have gotten it on the train. I think the time necessary for development of gonorrheal ophthalmia is less than Dr. Ray has stated. I have in mind a case that developed inside of twelve hours.

"In regard to vaginitis: Any vaginitis in little girls or grown women which is sufficiently strong to produce a violent ophthalmia is gonorrheal in origin. I will remind Dr. Ray of an acute case that we saw some time ago and afterwards took the child to St. Joseph's Infirmary. I had been treating the little girl, aged eight years, for four or five weeks for what was supposed to be simple vaginitis, the diagnosis based upon the surroundings of the family, their social condition, etc. She developed a gonorrheal ophthalmia of a very striking nature, the mother brought her to me twelve hours later, and and it was with great difficulty that the eyes were saved. A week or two later, through reliable sources I learned that the father had been suffering from an acute gonorrhea, and it is probable that the so-called simple vaginitis was in reality gonorrheal in origin. I should have determined this by finding the gonococci in the secretions.

"In order to discuss Dr. Mathews' case satisfactorily, we would have to know a great many things about which we now have little or no information. But I believe we are all agreed that the source of the infection had nothing to do with the rectal condition in the husband that the doctor was called upon to treat, or with the secretions following the operation."

Dt. T. C. EVANS: "I think Dr. Mathews has probably felt in his mind to criticise the oculists a little more severely than the c nditions demand. I can not believe that the woman had gonorrheal ophthalmia from the fact that these two oculists saw the case, and they were presumably good men, who understood their business, and they both recommended enucleation, which is not one of the remedies for gonorrheal ophthalmia. It may have been panophthalmitis. As to the source of the infection, we can not say anything positively about it."

